# Comorbidities of sarcoidosis

**DOI:** 10.1080/07853890.2022.2063375

**Published:** 2022-04-20

**Authors:** Claudio Tana, Marjolein Drent, Hilario Nunes, Vasilis Kouranos, Francesco Cinetto, Naomi T. Jessurun, Paolo Spagnolo

**Affiliations:** a Geriatrics Clinic, Medicine Department, SS Annunziata Hospital of Chieti, Chieti, Italy; b Department of Pharmacology and Toxicology, Faculty of Health, Medicine and Life Science, Maastricht University, Maastricht, The Netherlands; c ILD Center of Excellence, Department of Respiratory Medicine, St. Antonius Hospital, Nieuwegein, The Netherlands; d ILD Care Foundation Research Team, Ede, The Netherlands; e AP-HP, Hôpital Avicenne, Service de Pneumologie, Centre de Référence des Maladies Pulmonaires Rares de l'adulte, Université Sorbonne Paris Nord, Bobigny, France; f Interstitial Lung Disease Unit, Royal Brompton Hospital, National Heart and Lung Institute, Imperial College London, London, UK; g Rare Diseases Referral Center, Internal Medicine 1, Ca' Foncello Hospital - AULSS2 Marca Trevigiana and Department of Medicine - DIMED, University of Padova, Italy; h Netherlands Pharmacovigilance Centre Lareb, ‘s-Hertogenbosch, The Netherlands; i Respiratory Disease Unit, Department of Cardiac, Thoracic, Vascular Sciences and Public Health, University of Padova, Padova, Italy

**Keywords:** Adverse drug reactions, comorbidity, heart disease, multidisciplinary management, pulmonary hypertension, sarcoidosis, sarcoid-like reaction, treatment

## Abstract

Sarcoidosis is a heterogeneous disease, which can affect virtually every body organ, even though lungs and intra thoracic lymph nodes are almost universally affected. The presence of noncaseating granulomas is the histopathological hallmark of the disease, and clinical picture depends on the organs affected. Data about interaction between sarcoidosis and comorbidities, such as cardiovascular and pulmonary diseases, autoimmune disorders, malignancy and drug-related adverse events are limited. Several lung conditions can be associated with sarcoidosis, such as pulmonary hypertension and fibrosis, making it difficult sometimes the differentiation between complications and distinctive pathologies. Their coexistence may complicate the diagnosis of sarcoidosis and contribute to the highly variable and unpredictable natural history, particularly if several diseases are recognised. A thorough assessment of specific disorders that can be associated with sarcoidosis should always be carried out, and future studies will need to evaluate sarcoidosis not only as a single disorder, but also in the light of possible concomitant conditions.

**Key messages**
- Comorbidities in sarcoidosis are common, especially cardiovascular and pulmonary diseases.
- In the diagnostic workup, a distinction must be made between sarcoidosis-related complaints and complaints caused by other separate disorders. It can be very difficult to distinguish between complications of sarcoidosis and other concomitant conditions.
- The coexistence of multiple conditions may complicate the diagnosis of sarcoidosis, affect its natural course and response to treatment.

## Introduction

Sarcoidosis is a heterogeneous disorder affecting several organs and tissues, although the lungs and intrathoracic lymph nodes are the most common localizations of the disease. The histopathological hallmarks are non-necrotising granulomas that may have some distinctive features from other granulomatous disorders, though in some cases they can be indistinguishable [1]. It is generally accepted that sarcoidosis develops in individuals with a certain genetic background who are exposed to environmental/infectious antigens, which remain unknown. Certain drugs as well as other conditions, such as cancer, have been also associated with a granulomatous reaction indistinguishable from sarcoidosis [2,3]. Clinical picture is variable and depends on the organ(s) primarily affected, with non-specific symptoms of respiratory involvement (e.g. dry cough, dyspnoea, fatigue and chest pain) representing the most common clinical presentation [2,4]. The incidence of the disease is highest in Scandinavian countries (11.5 per 100,000), followed by United States (8–11 per 100,000), while the prevalence ranges from 1–5 per 100,000 in Japan, Taiwan and South Korea to 140–160 per 100,000 in Sweden and Canada. The highest mortality rate adjusted for age is observed in Black Americans [5]. An acute presentation followed by spontaneous resolution is observed in more than half of patients with sarcoidosis, whereas in approximately one third of patients, the disease takes a chronic course. Less than 10% of patients die from sarcoidosis with most fatal cases being due to advanced lung disease, followed by cardiac complications. Generally, patients with an acute onset as in Löfgren’s syndrome (e.g. bilateral hilar lymph adenopathy, erythema nodosum, fever and arthritis) have a favourable prognosis, whereas the presence of cardiac, neurologic, renal, and progressive fibrotic pulmonary involvement with respiratory failure is associated with increased morbidity and mortality as well as chronic course, and indicates the need for treatment [2]. The specific association and/or interaction with comorbidities such as cardiovascular and pulmonary disorders, autoimmune diseases, malignancy and drug-induced adverse events remains debated. Comorbidity is defined as a medical condition that is simultaneously present with another (or others) in a patient, either independent or related with it. In some cases, the causal relation between the two conditions can be easily identified, in others, the link may not be obvious until the disease becomes clinically overt [6]. Population-based cohort studies can be useful to explore the longitudinal exposure-outcome relations of a defined population. The impact of a specific comorbidity measured at baseline and over time is a good research strategy as it avoids confounding bias and allows to evaluate the outcomes [7]. The purpose of this narrative review was to analyse all population-based cohort studies aimed at investigating the interaction between sarcoidosis and specific comorbid conditions.

To this end, a literature search was conducted by searching public databases (PubMed, Scopus).

Search queries were:

1. Sarcoidosis AND Lungs OR respiratory system OR pulmonary disease;
2. Sarcoidosis AND Heart OR Cardiovascular system OR Cardiovascular risk OR Cardiovascular disease;
3. Sarcoidosis AND autoimmune system OR autoimmunity;
4. Sarcoidosis AND tumours OR neoplastic disease OR lymphoma OR haematologic disorder;
5. Sarcoidosis AND endocrine system OR thyroid disease OR diabetes mellitus OR diabetes OR obesity OR overweight;
6. Sarcoidosis OR Sarcoidosis-like reaction AND drugs OR therapy OR drug treatment OR cortisone therapy OR glucocorticoids OR methotrexate OR azathioprine OR leflunomide OR biological agents OR tumour necrosis factor-alpha inhibitors.

Duplicated papers or papers that were not adherent to the topic of the review were excluded.

## Lung comorbidities and pulmonary hypertension

### Pulmonary hypertension in patients with sarcoidosis

The exact prevalence of sarcoidosis associated pulmonary hypertension (SAPH) remains uncertain due to significant variability in diagnostic methods and criteria used as well as the populations studied. Overall, PH affects 1–6% of sarcoidosis patients [8], but it is much more frequent in advanced lung disease, reaching 73.8% of cases listed for lung transplantation [9]. In the study by Huitema et al., which has prospectively screened 399 consecutive patients from a Dutch cohort using transthoracic echocardiography (TTE) and confirmatory right heart catheterisation (RHC) in those with suspected PH, the prevalence of hemodynamically confirmed SAPH was 2.9% [10].

#### Mechanisms and classification

According to the European Society of Cardiology (ESC)/European Respiratory Society (ERS) task force and 6^th^ World Symposium, SAPH is placed in Group 5.2 along with other forms of PH deemed to multifactorial and/or unclear pathophysiologic mechanisms [11,12]. Although the main mechanism of SAPH is destruction of the distal capillary bed by pulmonary fibrosis and resultant hypoxia, there is a multitude of other pathophysiologic processes that may fit into all five categories of PH and often overlap [11,12].

The large majority of patients with SAPH have fibrotic lung disease. However, about one third of cases develop this complication in the absence of patent pulmonary fibrosis on chest radiography and a small subset has no apparent parenchymal disease [11]. Moreover, hemodynamics do not correlate well with spirometric parameters and hypoxaemia, and the degree of PH is sometimes “out of proportion” to functional abnormalities, thus suggesting the role of other pathophysiologic mechanisms [13–15]. These include specific pulmonary vasculopathy, locally increased vasoreactivity, portal hypertension due to liver involvement, extrinsic vascular compression, and left heart dysfunction. Other lung comorbidities associated with sarcoidosis can also cause PH, including obstructive sleep apnoea (OSA), and thromboembolic disease [8].

**Figure 1.**
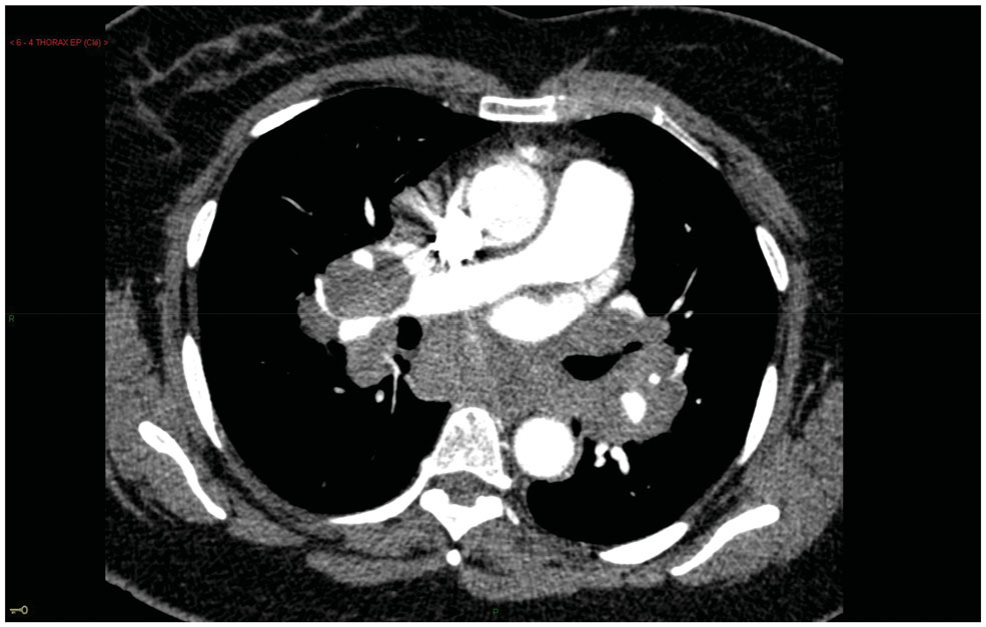
SAPH due to extrinsic vascular compression. CT scan shows compression of the right mediastinal pulmonary artery by enlarged lymph nodes. Note the increased diameter of main pulmonary artery as compared to aorta diameter.

In sarcoidosis, granulomatous infiltration of pulmonary circulation, which can be seen at all levels from large branches of pulmonary arteries (PAs) to pre-capillary arterioles and post-capillary venules, may contribute to the development of SAPH. Proliferative arteriopathy with plexiform lesions, the hallmark of group 1 pulmonary arterial hypertension (PAH), is rare in SAPH. A pulmonary veno-occlusive disease (PVOD)-like phenotype has been occasionally described [8]. SAPH may be related to extrinsic compression of the proximal PAs or veins by enlarged lymph nodes or fibrosing mediastinitis (Figure 1). This mechanism affects 5 to 21.4% of patients with SAPH [13,16].

Clinical myocardial involvement is observed in about 5% of sarcoidosis patients, and it can manifest as left ventricular (LV) systolic or diastolic dysfunction. Left heart dysfunction is probably underestimated in SAPH. In the study by Baughman et al. on sarcoidosis patients with persistent dyspnoea investigated by RHC, post-capillary PH was documented in 15.4%, which represented 28.6% of all SAPH cases, with only a minority having an impaired LV ejection fraction on TTE [17].

#### Diagnosis and management of SAPH

**Figure 2.**
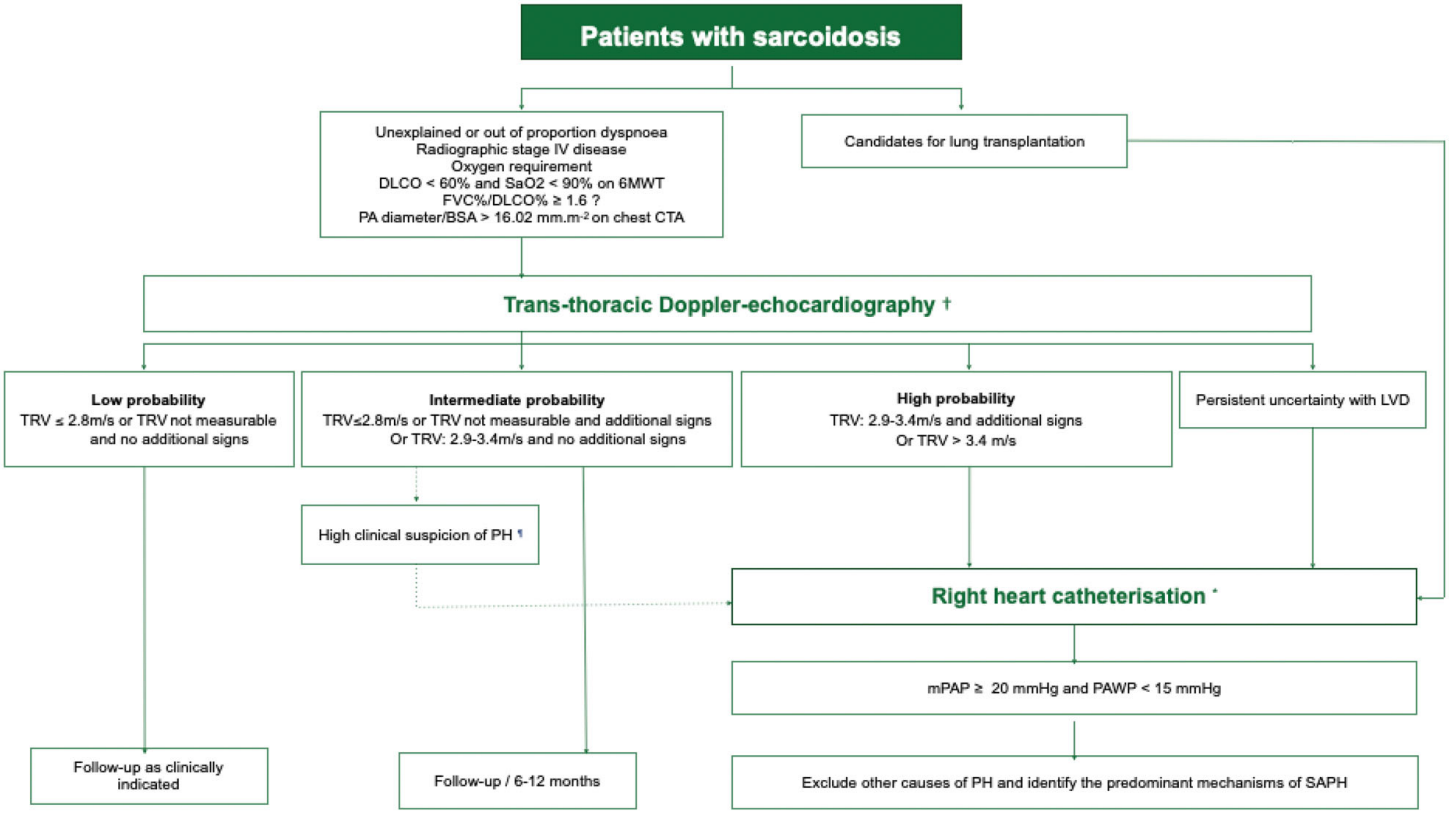
Screening and diagnosis of SAPH [8,11]. *Decision to proceed with right heart catheterisation should be discussed by a multi-disciplinary team with a sarcoidosis and a PH expert and take into account potential clinical consequences, and lung function. † Additional echocardiographic signs suggestive of PH [10]. ^¶^In cases with a high index of clinical suspicion, right heart catheterisation should be considered on a case-by-case basis. SAPH: sarcoidosis-associated pulmonary hypertension, 6MWT: six minutes’ walk test, FVC: forced vital capacity, DLCO: diffusing capacity for carbon monoxide, PA: main pulmonary artery, BSA: body surface area, CTA: computed tomography angiography, TRV: peak tricuspid regurgitation velocity, RHF: right heart failure; LVD: left ventricular dysfunction, mPAP: mean pulmonary arterial pressure; PAWP: pulmonary arterial wedge pressure, SAPH: sarcoidosis-associated pulmonary hypertension.

A statement endorsed by the World Association of Sarcoidosis and Other Granulomatous diseases (WASOG) has recently provided recommendations on SAPH diagnosis. A diagnosis algorithm is proposed in Figure 2 [8]. Although several clinical, functional and imaging features may raise suspicion of SAPH, there is no consistent single criterion that can adequately distinguish sarcoidosis patients with a high or low risk. Persistent or worsening dyspnoea in the absence of worsening parenchymal disease or restriction, or in the presence of worsening diffusing capacity for carbon monoxide (DLCO) should prompt consideration for SAPH [8]. Desaturation under 90% on six-minute walk test (6MWT) and DLCO <60% are associated with a twelve-fold and seven-fold increase in underlying PH, respectively [18]. Despite limited data in SAPH, forced vital capacity (FVC)/DLCO ratio >1.6, reflecting a disproportionately reduced DLCO for the degree of restriction, may be useful [8]. The Brain Natriuretic Peptide (BNP) or N-Terminal proBNP have a poor discriminative capacity for SAPH [19]. In the study by Huitema et al. PA diameter indexed to body surface on CT angiography showed the best positive and negative predictive values for detecting SAPH (70% and 93.2%, respectively), using a cut-off of 15.2 mm.*m*^−2^. The accuracy of this ratio was greater than that of PA diameter/aorta diameter ratio [20].

TTE is the most appropriate modality for the non-invasive assessment of suspected SAPH, with grading of the probability of PH as high, intermediate or low, based on both the tricuspid regurgitation velocity and other indirect measures [11,12]. Although RHC remains the gold standard for diagnosing SAPH, there is no consensus regarding which patients should undergo RHC [8]. The decision to proceed with RHC should take into account potential clinical consequences, in particular if PAH therapy is considered. RHC is reasonable in patients with a high probability of PH on TTE. In those with intermediate or inconclusive results and a high clinical suspicion of SAPH, RHC should be discussed on a case-by-case basis by a multi-disciplinary team with a sarcoidosis and a PH expert.

Once SAPH is confirmed, a comprehensive workup is intended to identify the predominant phenotype of SAPH in each patient [21], as it subsequently drives treatment approach [8].

Recommendations for management are provided in the WASOG statement on SAPH [8].

Supportive therapy includes supplemental oxygen in patients with resting or exertional hypoxaemia, diuretics as needed, and correction of comorbidities.

There is very limited data on the effect of anti-inflammatory treatment in SAPH, with variable results [13,16,22]. In patients with active parenchymal disease, it makes intuitive sense to control inflammation either before or in parallel to PH treatment. The residual activity may be best assessed by 18-fluorodeoxyglucose positron emission tomography (^18^FDG-PET), in particular in patients with fibrotic lung disease or extrinsic vascular compression by enlarged lymph nodes or fibrosing mediastinitis [13].

There is no consensus regarding which patients with SAPH should receive PAH-therapy, and which agents should be favoured [8]. Decision on PAH-therapy and follow-up should be made on a case-by-case basis by a multi-disciplinary team with a sarcoidosis and a PH expert [8].

Available data on the long-term efficacy and safety of PAH-therapy in SAPH are scarce, with a majority of retrospective cohorts [13–15], few prospective uncontrolled open label studies [23–25], and only two small size double-blind randomised placebo-controlled trials, one with bosentan, an endothelin receptor antagonist [26] and one with riociguat, a guanylate cyclase stimulator [27]. Overall, PAH-therapy improves pulmonary hemodynamics, but this benefit is not consistently observed in 6MWT, functional status or quality of life [8]. Although PAH-therapy is generally well tolerated in SAPH, worsening hypoxaemia due to ventilation/perfusion mismatch and shunting needs to be monitored. Patients with PVOD may develop pulmonary oedema under PAH-therapy.

Balloon angioplasty with or without stenting of focal proximal stenosis or compressions of pulmonary vessels may lead to significant improvement in hemodynamics and 6MWD [28].

Presence of PH reinforces the indication for lung transplantation in sarcoidosis patients, and patients with SAPH who have failed to respond to PAH-therapy and are otherwise eligible candidates should be referred for transplantation evaluation. SAPH does not seem to be associated with higher mortality after lung transplantation [29].

#### Prognosis and risk factors for mortality

PH is associated with high morbidity in sarcoidosis patients, as evidenced by substantially reduced exercise capacity and functional status [8], increased oxygen use, and need for caregiver assistance [9]. PH is an independent predictor of death, with a 8–10 fold increase in mortality compared to sarcoidosis patients without PH [9,16,17,30–32]. Median survival in patients with SAPH is between 4.2 and 6.8 years, with an estimated 3-year survival of 72–74% and 5-year survival of 55–62%, according to three large cohorts [13,15,32]. The risk of death of pre-capillary SAPH is more than 3-fold higher than that of SAPH with LV dysfunction [17].

Hemodynamic parameters do not seem to be associated with mortality in SAPH. In the international registry by Shlobin et al., DLCO < 35% and 6MWT distance <300 m were strong predictors of decreased survival [15]. Similarly, in the French registry by Boucly et al. including patients with severe SAPH, only 6MWT distance was independently associated with mortality [13].

### Pulmonary fibrosis with usual interstitial pneumonia pattern

**Figure 3.**
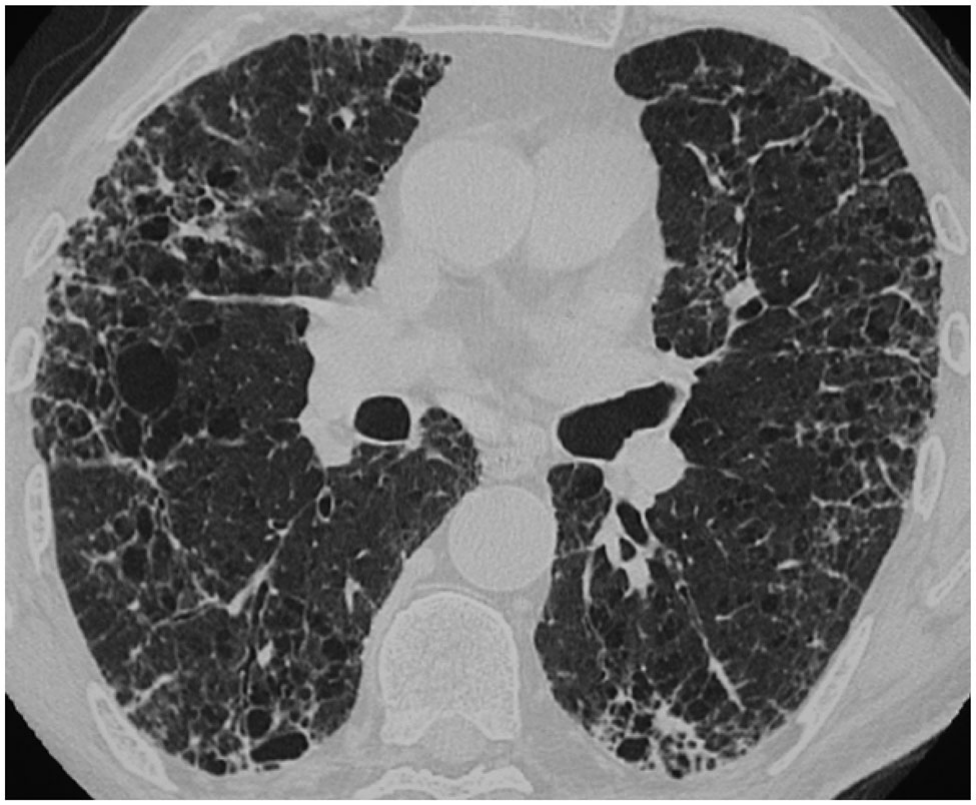
UIP-like comorbidity in a patient with sarcoidosis. CT scan shows a UIP-like pattern, with reticulations, bronchiectasis, and honeycombing with a basal and peripheral predominance in a patient with confirmed sarcoidosis.

Pulmonary fibrosis occurs in up to 20% of sarcoidosis patients and is thought to result from the granulomatous inflammatory process. Honeycombing can exist in fibrotic sarcoidosis but, in contrast to idiopathic pulmonary fibrosis (IPF), it usually predominates in the upper lobes and in a peri-bronchovascular location [33]. Interestingly a Usual Interstitial Pneumonia (UIP) pattern with basal and subpleural honeycombing has been observed on CT in a percentage of sarcoidosis patients (Figure 3), which has been confirmed in explant case reports and series showing isolated UIP pattern, including *fibroblastic foci*, the histological hallmark of UIP, or UIP with concomitant granulomas [34–36].

Collins et al. reported 25 patients with so-called « combined sarcoidosis and IPF » (CSIPF), as defined by clinical, radiographic, and histological features of both sarcoidosis and IPF [34]. The gender and race of patients with CSIPF and lone-IPF patients were similar (68% male and 84% Caucasian), as was survival, with a mean survival of 3.2 years in CSIPF and 3.6 years in lone-IPF [34]. However, it is currently unknown if CSIPF results from the *de novo* development of a second lung disease or if fibrotic sarcoidosis progresses to UIP pattern in genetically predisposed patients.

In contrast to IPF, pulmonary fibrosis in sarcoidosis may still be accompanied by active inflammation [37]. Experts agree that therapeutic goals of pulmonary fibrosis in sarcoidosis should focus on suppression of inflammation induced organ dysfunction, and on the avoidance of toxic effects of drugs [38]. So the usual anti-inflammatory strategy can be used including tumour necrosis factor inhibitors in the presence of granulomatous inflammation, which may be best evaluated by ^18^FDG-PET [37]. However, one should keep in mind that they may be ineffective as they have no impact on established irreversible fibrosis. Although the usefulness of antifibrotic dugs in the management of fibrotic sarcoidosis remains unknown, this may be proposed in patients with UIP pattern considering that CSIPF represents two combined disorders.

### Chronic pulmonary aspergillosis

#### Prevalence and risk factors

Chronic pulmonary aspergillosis (CPA) may be associate with pulmonary sarcoidosis (SA-CPA), with an estimated prevalence of 3–12% of cases [39], and sarcoidosis is the predisposing condition for CPA in 1.3% of patients with CPA [40]. SA-CPA mainly develops in patients with advanced pulmonary fibrocystic sarcoidosis, often with a bronchial distortion pattern associated with masses [31,41,42]. Masses, secondarily excavated, might be the nidus for aspergillus colonisation. Studies have implicated corticosteroids and immunosuppressive drugs in SA-CPA, as well as previous pneumothorax and smoking [41]. Interestingly, in the largest case-control study by Uzunhan et al. on 65 patients, a job with high-risk mould exposure was a significant risk factor for SA-CPA occurrence [42]. This finding highlights the importance for patients with fibrocystic sarcoidosis of taking precautions to protect themselves from mould exposure, including avoidance of gardening, spreading mulch or proximity to construction or renovation [43,44].

#### Diagnosis and treatment

About 37% of patients report haemoptysis at SA-CPA diagnosis, and 40% have weight loss [42]. The categorisation of SA-CPA [43,44] is difficult. However, the most frequent phenotypes of SA-CPA are chronic cavitary pulmonary aspergillosis (60%) and simple aspergilloma (37.8%), which may overlap, while chronic fibrosing pulmonary aspergillosis and subacute invasive aspergillosis are much rarer. Allergic bronchopulmonary aspergillosis has also been exceptionally described [42].

Bronchial arterio-embolization may be required for abundant or recurrent haemoptysis, with often the need for iterative interventions in SA-CPA [42]. Controversy remains regarding surgical CPA care, as peri-operative management is challenging (persistent air-leak, bronchopleural fistula, fungal spillage into the pleural space, and/or wound infection) [43,44]. Resection surgery should be proposed in strictly localised aspergillomas, which is a very rare condition in SA-CPA, and for severe bleeding uncontrolled by interventional radiology [43,44]. Many patients with SA-CPA are unsuitable for surgical cure due to extensive disease or poor respiratory function, and relapse is frequent. In the study by Uzunhan et al., 11 patients underwent resection surgery, mainly because of severe haemoptysis, with no peri-operative deaths, but 10 patients had a relapse after surgery, with a median delay ranging from six to 45 months, and four patients died during a longer follow-up [42]. Pre-operative and post-operative anti-fungal treatment should be considered in any complex situation.

Intracavitary amphotericin B instillation has been used successfully in some series [45]. There is only limited information in the literature on anti-fungal treatment in CPA, and no trial has specifically focussed in SA-CPA. Azoles, including itraconazole and voriconazole, are generally the preferred first-line drugs [43,44]. In the study by Uzunhan et al., anti-fungal treatment (voriconazole in 24 patients and itraconazole in six) showed a short-term benefit, as evidenced by a significant decrease in the maximal thickness of cavitation wall and pleura on CT after six to 12 months. However, CPA flare-up was frequent after treatment withdrawal [42]. Whenever possible, corticosteroids and immunosuppressive drugs should be reduced to control CPA.

SA-CPA is reported to have a high mortality rate in relatively ancient studies [39,46–50]. Combining four series showed 44 deaths out of 93 patients (47%), of which 12 (27%) died from massive haemoptysis [46,47,50]. In the study by Uzunhan et al., CPA did not seem to significantly affect survival. The survival of patients with SA-CPA was 73% and 61% at 5 and 10-year, respectively, which was not different from sarcoidosis controls without CPA. Mortality was mainly related to advanced pulmonary sarcoidosis, while at most 3 out of 27 deaths were attributable to CPA through massive haemoptysis. The independent predictive indicators of mortality at CPA diagnosis were PH, CPI (*p* = 0.004) and fibrosis extent [42].

### Other infections

According to a large nationwide Swedish study, serious infections are more common in sarcoidosis than in the general population, with an overall 1.8-fold increased rate of first hospitalised infection. Most detected infections were respiratory infections (bacterial or from unspecified organisms), urinary tract infections, sepsis, pyelonephritis, erysipelas, gastroenteritis, and colitis. The risk of serious infection in patients receiving immunosuppressive drugs is double than that of untreated patients, and it is maximal during the first two years of follow-up [51]. The risk is particularly high in patients treated with cyclophosphamide [52] or anti-tumour necrosis alpha (TNF-α) agents, and in those with bronchiectasis [53].

Sarcoidosis patients may be at risk for opportunistic infections. Unlike peripheral lymphocytopenia, glucocorticoids (GCs) are a major risk factor for opportunistic infections but the occurrence of such infections in untreated patients suggests more complex predisposing mechanisms. Aspergillosis, mycobacterial infection, cryptococcosis, and progressive multifocal leukoencephalopathy are the most common opportunistic infections [54]. Other infection comorbidities should be considered in the differential diagnosis. For instance, the risk of sarcoidosis is ∼8 fold higher among those who have a history of tuberculosis (TB) infection, and newly diagnosed sarcoidosis patients are at increased risk for TB. Other studies have correlated histoplasmosis and C. Acnes (formerly P. Acnes) to disease pathogenesis and should at least be considered among the differential diagnostic possibilities [51].

### Obstructive sleep apnoea

Poor subjective sleep quality and excessive daytime sleepiness are significantly higher in sarcoidosis patients than in the general population [55–57]. However to date, poor data is available on the frequency of OSA and its impact in sarcoidosis, and the mechanistic interactions between the two conditions are unclear and controversial. Similar to other ILD, progressive lung restriction may influence the patency of the upper airway and hence increase its collapsibility [56,58,59].

OSA may also be due to the granulomatous infiltration of the upper airway by sarcoidosis, as suggested in a study that found an association between OSA and lupus pernio [60]. Other potential causes include GC-induced obesity and sarcoidosis small fibre neuropathy [57,61].

The prevalence of OSA is about 60% [59], with reported rates ranging between 17% and 88.2% in small-sample size studies focussing specifically on sarcoidosis, or in cohorts of patients with various ILDs [56,60–63], which seems higher than in matched control groups [60], but remains lower than in IPF [59]. US Hispanics sarcoidosis patients may be at higher risk of OSA than non-Hispanics [64], and sarcoidosis patients with lower incomes may experience more frequently OSA as a GCs-related comorbidity [65]. Although frequent in sarcoidosis, fatigue should exclude the possibility of OSA. Hypercapnia is unusual in sarcoidosis in the absence of severe pulmonary dysfunction and should prompt evaluation for OSA [58].

In the study by Mari et al., which has systematically investigated 68 unselected sarcoidosis patients, OSA affected 88.2% of them. OSA was mild (5 ≥ apnoea/hypopnea index (AHI) > 15) in 36.8%, and moderate-to-severe (AHI ≥ 15) in 51.5% of cases [62]. This surprisingly high prevalence may suffer from a selection bias since patients were recruited in a referral centre for ILD. Furthermore the overnight sleep study was performed using a portable device instead of supervised in-laboratory polysomnography. Male gender, body mass index, Scadding stage, total lung capacity and FAS-score were predictors of AHI severity while sleepiness and GCsuse for more than 3 months at baseline were not [62]. Amongst the 20 patients treated with CPAP and evaluated at 3 months, FAS and Epworth Sleepiness Scale (ESS) scores improved significantly. Compliance to treatment was good in 65.0% of cases [62].

Other obstructive pulmonary disorders which may enter into differential diagnosis with sarcoidosis are chronic obstructive pulmonary disease (COPD) and asthma, which are common comorbid conditions, and sarcoidosis may predispose to them [2].

## Comorbid cardiovascular disease and CV risk in patients with sarcoidosis

### Cardiovascular risk and comorbidities in sarcoidosis

Cardiovascular disease (CVD) is one of the leading causes of morbidity and mortality worldwide in the general population and the second most frequent cause of death in patients with sarcoidosis [66]. The term “cardiovascular risk” is defined as the probability of developing CVD within a defined period of time, taking into account several risk factors simultaneously. It is therefore critically important to design optimal strategies in identifying patients at high risk of developing CVD. This would enable the development of appropriate primary preventative interventions.

### General cardiovascular risk assessment

In the general population, smoking, hypertension, diabetes and hyperlipidaemia substantially increase the risks for cardiovascular complications. The effect of these “traditional” cardiovascular risk factors may be amplified in sarcoidosis patients since they are also associated with the use of immunosuppression. Ischaemic heart disease (IHD) was reported as underlying cause of death in French decedents (2002–2011) in 2%, when sarcoidosis was listed as a non-underlying cause of death. When sarcoidosis was listed as the underlying cause of death, IHD was reported in 7% as non-underlying cause of death [67]. There is evidence to support increased risk of atherosclerosis in sarcoidosis population as well as other immune-mediated diseases [68–71]. A Population-Based Retrospective Cohort Study in Minnesota found that the prevalence of CVD was not significantly different between sarcoidosis patients and age/gender matched comparators before index date [72]. However, after index date, the risk of incident CVD was significantly elevated among sarcoidosis patients [HR: 1.57, (95% CI, 1.15 − 2.16)] even after adjusting for the traditional cardiovascular risk factors [HR: 1.65, (95% CI, 1.08 − 2.53)]. Rossides et al. found a slightly increased risk of acute myocardial infarction (AMI) associated with sarcoidosis. However, a 40% higher risk associated with sarcoidosis was observed in a subgroup of patients who received immunosuppressant treatment around sarcoidosis diagnosis likely due to more severe disease [73]. The granulomatous inflammation and the related oxidative stress may significantly affect lipid metabolism and contribute to CVD pathogenesis. Sarcoidosis patients have been found to have overexpression of proteins involved in HDL and fatty acid metabolism, which can increase the risk of developing atherosclerosis. In addition, the chronic steroid treatment in a significant proportion of sarcoidosis patients is known to result in poorer control of blood pressure and diabetes, obesity and hyperlipidaemia [74]. It becomes clear that all sarcoidosis patients and especially those on immunosuppressive treatment should undergo “traditional” cardiovascular risk assessment both at baseline and at least when they report cardiac symptoms. Regular review of the blood pressure, diabetes and lipid profile control seems essential especially for those on treatment. However, there is no convincing evidence to support the need for any preventive treatment in this setting.

### Cardiac sarcoidosis risk assessment

In the sarcoidosis population, the development of myocardial involvement is a common cause for major cardiovascular events including major arrythmias, heart failure and even sudden cardiac death. The largest case-controlled study to date (the ACCESS [A Case Control Etiologic Study of Sarcoidosis] study) had originally reported a prevalence of 5% of cardiac sarcoidosis (CS) [75]. In the last two decades, the wider use of advanced imaging modalities [Cardiac Magnetic Resonance Imaging (CMR) and Flurodeoxyglucose Cardiac Positron Emission Imaging (FDG-PET)] as well as the increased awareness of the disease revolutionised the diagnosis and management of CS. As a result, an increased prevalence of CS has been reported over the years, which is similar to the one reported in autopsy studies [76,77].

The clinical presentation of CS is highly variable as with all forms of sarcoidosis. It can range from an incidentally ECG or echocardiographic abnormality without any symptoms to heart failure and sudden cardiac death depending on the location of granulomatous inflammation. Advanced atrioventricular block (and other conduction abnormalities) and ventricular tachycardia are the most common ECG abnormalities, while supraventricular arrhythmias may or may not be related with CS. Sudden cardiac death is well reported in the sarcoidosis population even in patients with preserved left ventricular function. In advanced forms of the disease, heart failure develops from extensive myocardial fibrosis and/or inflammation.

The diagnosis of CS is often challenging. The lack of a diagnostic gold standard has been highlighted and several diagnostic guidelines have been published to address this issue [78–80]. Endomyocardial biopsy would be the gold standard diagnostic tool, but this is associated with low diagnostic yield due to the patchy nature of the disease and therefore often avoided outside expert centres [81]. In essence, all guidelines recommend a multi-disciplinary approach when reaching a clinical diagnosis of CS. A multi-disciplinary review of clinical information, ECG abnormalities, echocardiographic abnormalities and advanced imaging modalities is necessary in order to reach a confident and accurate clinical diagnosis of CS. Accordingly, a confident clinical CS diagnosis may not require histological confirmation. The outcomes in one study between CS patients diagnosed with endomyocardial biopsy versus clinical and imaging criteria were similar [82].

The role of CMR is invaluable for the diagnosis and management of CS. CMR is the gold standard tool in providing an accurate assessment of the myocardial structure and morphology as well as tissue characterisation. The detection of late gadolinium enhancement (LGE) on CMR in specific patterns compatible with CS is considered a hallmark of CS. CMR can also detect edoema. T2- weighted sequencing, which is associated with inflammatory activity, has prognostic implications and can be reflective of disease regression during treatment [78]. CMR is currently recommended as the first diagnostic tool in all sarcoidosis patients with cardiac symptoms and/or ECG abnormalities, as supported by a systematic review of all available literature [83]. In addition, CMR plays a crucial role in the risk stratification and prognosis. The presence of LGE is an independent predictor of major cardiovascular events even in the general sarcoidosis population [84–87]. A meta-analysis of 694 patients has shown that sarcoidosis patients with CS (defined by LGE on CMR) had an approximate 11-fold higher risk of cardiovascular mortality and 20-fold higher risk of ventricular arrhythmias when compared to non-CS patients [83].

FDG-PET scan is useful to detect the presence of active myocardial inflammation, and is usually performed if CMR shows evidence of cardiac involvement or there is a high degree of clinical suspicion of CS [86]. Despite this, the recommendation for serial PET scans to monitor cardiac sarcoidosis is controversial. For instance, it would not be compensated by most insurance agencies in US while in Europe FDG-PET is used to follow-up patients with sarcoidosis and to monitor response to treatment [88]. A retrospective study in a cohort of 107 patients showed that 45% of patients had to be reclassified regarding the level of confidence of CS diagnosis when CMR and FDG-PET were integrated highlighting the importance of a multidisciplinary approach in diagnosis of CS [89]. The presence of active myocardial inflammation has been associated with adverse outcomes and therefore is a clear target of treatment in CS [90]. FDG-PET is critical in disease monitoring and is frequently repeated six to 12 months after the introduction of immunosuppressive treatment to evaluate the degree of response to treatment.

Unfortunately, there is no optimal strategy developed to screen the sarcoidosis population for cardiac involvement. The American Thoracic Society clinical practice guidelines recommend that all sarcoidosis patients should be interrogated for cardiac symptoms including palpitations, episodes of syncope and pre-syncope as well as (typical or atypical) chest pain at every outpatient clinic visit (if possible) and have a 12-lead ECG at baseline [91]. Nonetheless, previous studies have shown that the diagnostic yield of such approach is rather low (sensitivity: 69% and specificity:46%, AUC:0.573) [84]. Therefore, a low threshold for the performance of advanced imaging modalities should be considered in the appropriate clinical setting. The role of emerging echocardiographic techniques (speckle tracking) as well as existing biomarkers (high sensitivity troponin and B-natriuretic peptide) in addition to the current strategies have not yet been validated.

Management of CS is focussing on a) eliminating the existing myocardial inflammation, b) supporting the myocardial function when remodelling has occurred and c) preventing future life-threatening arrhythmias. The 10-year survival in CS was recently reported at 90% [92]. Core measures of the cardiac structure (left ventricular ejection fraction), extent of LGE on CMR and possibly active myocardial inflammation seem to be the determinants of outcome in all published series. ICD implantation is strongly recommended in patients presenting with sustained ventricular arrhythmias or those with left ventricular ejection fraction <35% despite medical therapy [78]. Sarcoidosis patients who need a permanent pacemaker or had inducible sustained VT or have extensive LGE on CMR may also benefit from ICD implantation after multi-disciplinary review of their data [93].

## Association with autoimmune disorders

### Sarcoidosis and specific autoimmune diseases

The association between sarcoidosis and autoimmune disorders has been described in several case reports and in large cohorts studies [94–96]. Considering that sarcoidosis itself has been suggested for a classification as a specific autoimmune disorder [97], the association is not surprising, as clustering is a classical feature of autoimmune diseases [98]. In 2017 a retrospective study on 1237 sarcoidosis patients from Taiwan demonstrated a higher risk of autoimmune comorbidities in comparison to age- and sex-matched control subjects, with a prevalence of 17.6% in sarcoidosis patients. In particular, autoimmune thyroid disease, Sjögren’s syndrome (SjS) and ankylosing spondylitis presented the highest Odds Ratios, with thyroid disease associated to the male and SjS to the female gender [95]. The association with autoimmune thyroid disease had already been described, both under the sarcoidosis and under the thyroiditis point of view [98–100]. This association will be addressed in the metabolic section of the present review. Also, an overlap between sarcoidosis and connective tissue diseases (CTDs) had been extensively reported [101]. Accordingly, the presence of antinuclear antibodies has been found to be significantly higher in sarcoidosis patients than in healthy controls, despite their pathogenetic and prognostic relevance being still undefined in absence of the specific criteria for a diagnosis of CTD [102,103]. Moreover sarcoidosis has been reported as co-existing with systemic granulomatous vasculitides, as Takayasu arteritis [104].

A more recent study on a European cohort of 1737 patients found 16% of patients presenting at least one associated immune-mediated disease, with a frequency almost 2-fold higher than in the general population [94]. This sub-cohort received the diagnosis of sarcoidosis at an older age and, unsurprisingly, presented a higher prevalence of women. The strongest association in comparison to the general population was found with familial Mediterranean fever, primary biliary cholangitis, haemolytic anaemia, autoimmune hepatitis, antiphospholipid syndrome, immune thrombocytopenia, SjS, systemic sclerosis, ankylosing spondylitis, and psoriatic arthritis. Again, autoimmune thyroiditis was the most common autoimmune finding, but without difference compared to the reference population. In terms of gender association, eosinophilic digestive disease was the more relevant in women while immune thrombocytopenia, systemic sclerosis and autoimmune hepatitis in men [94]. Table 1 shows the prevalence of autoimmune diseases that were detected in the two largest cohorts that recently investigated the association between sarcoidosis and autoimmune conditions.

**Table 1.** Comparison of prevalence of autoimmune diseases (AID) detected in the two largest cohorts recently investigating the association between sarcoidosis and AID.

Brito-Zeron *et al.* [94]	*n* = 1737	Wu *et al.* [95]	*n* = 1237
AID	Frequency	AID	Frequency
All associated AID	283 (16.3%)^a^	All associated AID	218 (17.6)^a^
Autoimmune thyroid disease	94 (5.4%%)	Autoimmune thyroid disease	143 (11.6)^a^
Sjogren’s syndrome	31 (1.7%)^a^	Sjogren’s syndrome	19 (1.54)^a^
Ankylosing spondylitis	13 (0.74%)^a^	Ankylosing spondylitis	45 (3.64)^a^
Systemic lupus erythematosus	7 (0.4%)^a^	Systemic lupus erythematosus	6 (0.49)
Myasthenia gravis	1 (<0.1%)	Myasthenia gravis	3 (0.24)
Systemic sclerosis	8 (0.46%)^a^	Systemic sclerosis	1 (0.08)
Psoriasis	29 (1.67%)	Psoriasis	12 (0.97)
Rheumatoid arthritis	9 (0.52%)	Rheumatoid arthritis	2 (0.16)
Inflammatory myopathies	2^a^	Dermatomyositis	2 (0.16)
Vitiligo	3 (0.17%)	Vitiligo	2 (0.16)
Alopecia aerata	c	Alopecia aerata	5 (0.40)
DM type 1	10 (0.57%)	DM type 1	0
Multiple sclerosis	8 (0.46%)^a^	Multiple sclerosis	0
Primary biliary colangitis	9 (0.52%)^a^	Primary biliary colangitis	c
Celiac disease	7 (0.4%)	Celiac disease	0
Psoriasic arthritis	5 (0.29%)^a^	Psoriasic arthritis	c
Antiphopsholipid syndrome	6 (0.34%)^a^	Antiphopsholipid syndrome	c
Ulcerative colitis	4 (0.23%)	Inflammatory bowel diseases	0
Autoimmune hepatitis	3 (0.17%)^a^	Autoimmune hepatitis	0
CVID	10 (0.57%)^a^	CVID	c
Immune thrombocytopenia^b^	9 (0.52%)^a^	Immune thrombocytopenia	c
Haemolytic anaemia^b^	3 (0.17%)^a^	Haemolytic anaemia	c
Familial Mediterranean Fever	1 (<0.1%)^a^	Familial Mediterranean Fever	c

^a^Increased compared to normative population/controls of the same study.

^b^Autoimmune cytopenia’s are a classical feature of CVID, but the study does not specify if and in which percentage immune thrombocytopenia or haemolytic anaemia occurred in concomitance with a diagnosis of CVID.

^c^Not detected in cases and not investigated in control group.

0 = not detected in cases but investigated and eventually detected in control group.

### Differential diagnosis and impact of treatments

Interestingly, the above mentioned two large cohorts, of similar size but of Eastern and Western origin, strongly agree in indicating that around one out of six sarcoidosis patient may present an associated immune-mediated complication [94,95]. When considering that SjS syndrome and ankylosing spondylitis are listed between the most common in both populations, it is easy to understand how overlapping symptoms (e.g. sicca syndrome or joint pain) and laboratory parameters (as polyclonal hypergammaglobulinemia) of co-existing diseases might delay the second diagnosis when the first one has already been established. The same might happen with certain organ-specific autoimmune diseases, as primary biliary cholangitis or autoimmune hepatitis. Other reported aspects possibly impacting on the differential diagnostic process include a trend towards higher number of extrathoracic organs involved in those sarcoidosis patients presenting an associated immune-mediated disease as well as eventual treatments that, at least in case of systemic autoimmune diseases, may somehow mask part of the clinical features of sarcoidosis [95]. It is also worth mentioning that drug-related sarcoidosis or sarcoidosis-like reactions have been reported in patients treated with immune-modulators, both PD1 inhibitors used in cancer treatment and anti-TNF or anti-IL6 drugs used in the treatment of CTDs and other systemic autoimmune diseases [105–107]. This suggests that the onset of the granulomatous disease might take advantage from the impact of targeted therapies on an already immune-dysregulated micro-environment, despite the same drugs being instead successfully used in the treatment of refractory sarcoidosis in a different biological context. Of note, in the Taiwan cohort the diagnosis of autoimmune diseases tended to be established after that of sarcoidosis, despite a cause-effect relationship having not been specifically investigated [95]. Similarly, it remains to be established whether the use of specific drugs for the treatment of the granulomatous disease might influence the occurrence of certain autoimmune diseases.

### Sarcoidosis and primary or acquired immune dysregulation diseases

It is interesting to note that the authors of the above cited Spanish study used the term “immune-mediated” rather than autoimmune diseases, including in the list the two most common primary antibody deficiencies of the adulthood, namely Common Variable Immunodeficiency (CVID) and selective IgA deficiency. CVID, in particular, was found as the immune-mediated disease with the highest degree of association with sarcoidosis [94]. The definition of CVID as an immune-mediated condition associated to sarcoidosis might be questionable, since CVID is a primary immunodeficiency characterised by recurrent infections, immune-dysregulation (leading to autoimmune cytopenia’s and other immune-mediated diseases) and cancer. However, a granulomatous disease resembling sarcoidosis has been described in up to 15–25% of patients affected by CVID [108,109]. It is important to mention that the occurrence of systemic sarcoidosis/sarcoidosis-like disease has also been described in other contexts of immunodeficiency and chronic infection-driven immune stimulation. Sarcoidosis may indeed occur, often as part of a more complex picture, in subjects with a chronic human immunodeficiency virus (HIV) or hepatitis C virus (HCV) infection. A study investigating the epidemiology of autoimmune and inflammatory diseases in a French nationwide cohort of HIV patients found sarcoidosis as one of the most prevalent inflammatory and autoimmune diseases (IAD) [110,111]. In particular, of the 1001 patients developing an IAD concomitantly or after HIV diagnosis, 31 developed sarcoidosis, of which 7 presenting HCV co-infection [111]. In a large series of 1020 patients with systemic autoimmune diseases associated with HCV infection from the HISPAMEC Registry, 28 patients were found to be affected by sarcoidosis, despite chronic HCV infections being more known mainly for its association with cryoglobulinemia and lymphoproliferative diseases [112].

In conclusion, the occurrence of systemic granulomatous disease in the context of immunodeficiency, autoimmunity and immune dysregulation, chronic infection-driven immune stimulation, as well as after administration of monoclonal antibodies and small molecule inhibitors during the treatment of other autoimmune diseases deserves further mechanistic investigations, highlighting once more the complexity of the pathogenetic pathways underlying sarcoidosis granulomatous inflammation.

## Sarcoidosis and risk of malignant tumours

An association between sarcoidosis and malignancy has been suspected for a long time. Brincker initially hypothesised the importance of this comorbidity in 1972 by observing an association between sarcoidosis cases and lymphomas [113]. The association has been a matter of discussion for several years and current evidence is controversial. The term "sarcoidosis-lymphoma syndrome" was coined for all the cases where a temporal association of lymphomas following sarcoidosis was documented [114].

### Association with lymphoproliferative malignancies

A continuous proliferation of lymphocytes associated with an increased mitotic activity, especially in the chronic active form of sarcoidosis, rather than the self-healing type, has been postulated as the main potential mechanisms of malignant risk [115]. Brincker observed that lymphoproliferative and myeloproliferative disorders tend to appear in 67–76% of cases more than 12 months after the onset of sarcoidosis, with a median time of three years [116]. A large retrospective study, which included patients from the Swedish Inpatients Register (1964–1994) found a doubled risk of non-Hodgkin lymphoma during the first ten years of follow-up [117]. These data were in contrast with the results of Seersholm et al., who followed a large cohort of patients for a median of 25 years and did not find cases of lymphomas over the observation period [118], and with the Danish study of Rømer *et al*, who followed more than 500 sarcoidosis patients for a long period and similarly did not find a significantly increased incidence of malignant lymphoma (Observed vs Expected -O/E- ratio 1.25, 95% CI 0.02–6.95) [119]. These conflicting results were in part explained selection bias and misclassification. Some data were indeed collected from old registries and some diagnoses of lymphomas were not biopsy-proven, (as in the study by Brincker et al.) [120]. Further studies have reinforced the risk of lymphoproliferative disorders in sarcoidosis patients. In particular, the Swedish cohort study, which enrolled over 10,000 patients who were hospitalised for sarcoidosis between 1964 and 2004, has documented an increased risk of malignancy (40% excess incidence in 1,045 patients), particularly non-Hodgkin's lymphoma and leukaemia [121]. Recently, a population-based cohort study analysed 3,892 biopsy-proven sarcoidosis patients compared to 38.920 population-controls matched for age and gender, and observed an increased risk of haematologic cancers (hazard ratio - [HR]- during the first and after 2 years of follow-up: 2.71 (95% CI: 1.18–6.25) and 2.12 (95% CI: 1.29–3.47) respectively. The risk persisted also after 10 years of follow-up (risk difference: 0.56% (95% CI: 0.11%-1.01%) [122].

### Association with other neoplastic conditions

Sarcoidosis has been associated with an increased risk of other tumours, not only lymphoproliferative disorders. Uncontrolled T-cell proliferation, chronic inflammation with reduced ability to eliminate toxic antigens, reduced function of myeloid dendritic cells (mDC) and of abnormal tumour immune surveillance have been postulated as potential mechanisms [120]. The overproduction of inflammatory cytokines and interleukins, which is commonly observed in the active form of sarcoidosis, might amplify the angiogenesis whereas the release of TNF- α from alveolar macrophage may induce cellular growth and tissue remodelling, all factors that might increase the risk of neoplastic proliferation [123]. Some studies have found an increased risk of tumours in the lungs, gastrointestinal and genitourinary system, skin, breast and thyroid glands [118,124–126]. However, these results are in contrast with previous data that had not found a significant increase of the hazard [127]. In the population-based cohort study of Faurschou *et al*, the risk of invasive solid tumours was high only during the first 2 years of follow-up, while persisted after ten years in patients with non-melanoma skin cancers [122]. Some authors suggest that sarcoidosis might contribute to worsening the prognosis rather than increasing the risk of disease development [125,128].

Of note, lesions mimicking sarcoidosis may also occur in patients with malignancies. Most recently, these lesions were reported in the context of immune checkpoint inhibitor treatment (e.g. for metastatic melanoma) [129]. This sarcoid-like response to cancer may be beneficial to mitigate disease spread, but may also cause harm. Thus, this represents a challenging comorbid association to manage.

## Metabolic conditions and other comorbidities

Diabetes mellitus, obesity, and thyroid diseases are among the most commonly reported metabolic comorbidities in patients with sarcoidosis.

### Type 2 diabetes

Several studies have shown that type-2 diabetes (T2D) is more prevalent in patients with sarcoidosis compared with age- and sex-matched controls [130–133]. However, to what extent the increased risk of T2D is accounted for by glucocorticoid (GC) use is unclear. In order to clarify this, Entrop and co-workers performed a large cohort study that included untreated and GC-treated sarcoidosis patients identified from the Swedish National Patient Register (*n* = 5,754) and general population (*n* = 61,297) matched by age, sex and region of residence [134]. The T2D rate was higher in both the GC-treated (12.7 per 1,000 person-years) and untreated (7.7 per 1,000 person-years) sarcoidosis groups compared with the general population (5.5 per 1,000 person-years), which translated to a risk of T2D 44% higher in untreated (HR: 1.44) and over two times higher in GC-treated sarcoidosis patients (HR: 2.44) compared with the general population. The risk was highest in males who received GCs at sarcoidosis diagnosis (15.0 per 1,000 person-years) and older patients. The presence and severity of metabolic disorders have also been suggested to impact on the severity of pulmonary sarcoidosis. In a retrospective cohort study, 220 sarcoidosis patients were categorised into two cohorts - diabetic (*n* = 66) and non-diabetic (*n* = 154) - and multiple metabolic factors, including body mass index (BMI), lipid status and glycemic control, were compared between the two cohorts [135]. Compared to non-diabetics, diabetic patients had lower total lung capacity (TLC) and diffusing capacity of the lung for carbon monoxide (DL_CO_). In addition, female, non-smoker and morbidly obese (BMI ≥35) diabetic patients had significantly lower DL_CO_ than their non-diabetic counterparts, suggesting metabolic disorders may be an independent risk factor for worse pulmonary sarcoidosis.

### Obesity

The relationship between obesity and sarcoidosis is unclear. Indeed, obesity can be both a potential contributing factor for and a consequence of sarcoidosis, particularly in patients on chronic GC treatment [136]. Yet, it is biologically plausible that obesity may influence the occurrence of sarcoidosis. Adipose tissue consists of several types of innate and adaptive immune cells [137], which with obesity undergo a shift towards a T-helper 1 proinflammatory phenotype [138,139].

In obese individuals, immune cells in the lung have been hypothesised to undergo a similar switch, possibly in response to the changes occurring in the adipose tissue [140].

The relation between obesity and sarcoidosis was initially explored by Gvozdenic et al. [141] who assessed the impact of high BMI on fatigue, dyspnoea, health status and spirometry in 184 sarcoidosis patients and 184 sex- and age-matched healthy controls. Patients with sarcoidosis were more likely to be overweight or obese, had higher fatigue scores, lower baseline dyspnoea index and forced expiratory volume in one second (FEV)/forced vital capacity (FVC) values, and poorer self-reported health status. A study of 75,000 women enrolled in the Danish National Birth Cohort explored the association between BMI (obesity) and 43 autoimmune diseases during 11 years of follow-up [142]. Obese women were at increased risk of developing sarcoidosis (HR: 3.59), type-1 diabetes (HR: 2.67) and psoriasis (HR: 2.16). The association between obesity, weight gain and incident sarcoidosis was assessed in a follow-up study of 59,000 Black women in a follow-up period between 1995 and 2011 [143]. In this latter study, it was found that obesity at baseline was associated with an increase of sarcoidosis incidence of 42% (incidence rate ratio (IRR): 1.42), whereas a weight gain of ≥30 kg between age of 18 years and baseline was associated with an increased incidence of 47% (IRR 1.47) [143]. Dumas et al. confirmed the association between obesity and sarcoidosis in white American women in a 24-year follow-up study [144]. It was found that obesity was associated with an increased risk of sarcoidosis of approximately 70% (HR: 1.74). Moreover, BMI at age 18 years and cumulative weight gain of ≥25 kg appeared to be associated with an IRR of sarcoidosis as well [144].

### Thyroid disease

Sarcoidosis shares a number of clinical features with several systemic and organ-specific autoimmune diseases [145], and some have hypothesised that sarcoidosis itself may be an autoimmune disorder [97]. A possible association between sarcoidosis and autoimmune thyroid disorders was initially suggested by anecdotal reports [146–148]. Sarcoidosis patients have also been shown to have higher levels of thyroglobulin antibodies and/or thyroid peroxidase antibodies compared to control subjects [149], although the pathogenetic role of these antibodies in the association between sarcoidosis and autoimmune thyroid disease is unknown [146]. The exact prevalence of hypothyroidism in sarcoidosis is unclear. A nationwide Taiwanese study that included 1,237 sarcoidosis patients and 4,948 age- and sex-matched controls found that around 12% of patients, mostly females, had an autoimmune thyroid disorder [95]. More recently, a large national registry-based study in the U.S. investigated the prevalence, clinical characteristics and impact of hypothyroidism among sarcoidosis patients (*n* = 3,835) who responded to the Sarcoidosis Advanced Registry for Cures Questionnaire [150]. Fourteen percent of patients, mainly middle-aged white women, self-reported hypothyroidism, a prevalence significantly higher than that reported in the general population in the U.S. [151], but similar to that observed in other smaller cohorts in Italy [98] and Greece [152]. Notably, hypothyroid patients were more likely to have multiorgan disease and more comorbid conditions, including obesity, sleep apnoea, chronic fatigue syndrome and depression, highlighting the importance screening for hypothyroidism in patients with sarcoidosis.

## Drug-induced comorbidities in sarcoidosis

As with many conditions, drug-induced damage from pharmacotherapy can occur in sarcoidosis patients too. This may be related to the pharmacotherapy of sarcoidosis itself or of certain comorbidities. These drug-induced or iatrogenic conditions can be hard to recognise as the clinical manifestations and/or symptoms might be quite similar to sarcoidosis-associated symptoms.

### Drug-induced comorbidities associated with pharmacotherapy of sarcoidosis

Current sarcoidosis treatment standards are based on low-quality evidence, and aim to suppress granulomatous inflammation and restore organ function, avoiding ARDs [38]. A consensus statement from sarcoidosis experts endorses GCs as the first line treatment for symptomatic sarcoidosis patients [38]. Methotrexate (MTX), azathioprine (AZA), leflunomide, and hydroxychloroquine (HCQ) are considered as second-line drugs. Biologics, such as TNF-α inhibitors, represent the third-line treatment. This approach is currently reserved for patients who do not respond to first- or second-line treatment or suffer from intolerable ADRs [2,38,153].

Prolonged therapy with GCs is associated with significant ADRs, such as weight gain, fatigue and muscle weakness (see Table 2) [154]. Moreover, use of higher doses of GCs has been linked to increased number of non-sarcoidosis-related emergency department visits compared to patients with lower cumulative GC exposure [155]. Harper et al. showed that new steroid-related comorbidities and poor outcomes can be predicted by low income in US patients with sarcoidosis, following by current or past medication use, higher age and longer duration of symptoms [65]. Moreover, individuals with sarcoidosis - especially those on treatment - experience a reduction in work ability that may persist for up to five years after diagnosis [156]. In line with previous studies, Drent et al. showed that the most important ADRs related to prednisone use were weight gain and increased appetite [157]. This increases - among others - the risk of other co-morbidities such as diabetes, exercise limitations, OSA, and fatigue. In this regard it is important to realise that for instance fatigue can be associated with sarcoidosis, but can also be caused by its treatment with GCs [57,158]. Therefore, it should be recognised as GC-associated fatigue and not strictly sarcoidosis-associated fatigue [159]. It has been observed also that GC use, even at dosage lower than < 5 mg/die, is associated with an increased risk for CVDs in immune-mediated diseases [160]. Therefore, it is important to regularly consider how to reduce the ADRs of GCs. As there is considerable variation in individual requirements for GC replacement, the balance between over- and under-replacement of GCs is a significant clinical challenge [161]. Unfortunately, no systematic record of ADRs, neither in general nor in sarcoidosis, exists. Systematic gathering of information about the patient’s phenotypes as well as patient’s genetic profile (including genes involved in drug metabolism) would help to identify those patients more prone to develop certain drug-induced adverse effects and co-morbidities [162]. This information has the potential to guide a personalised drug prescription. Moreover, for patients requiring long-term GC-treatment new strategies are needed to optimise patient management, including lifestyle changes, physical therapy, and dietary guidance [2,163]. Future studies will need to explore the mechanisms that cause GC harmful effects and to identify those patients on higher risk to develop them [154].

**Table 2.** Summary of clinical relevant adverse drug reactions (ADRs) of glucocorticoids and low-dose methotrexate organised by organ system affected, as well as sarcoidosis-associated morbidity [2].

System affected	Glucocorticoid ADRs	Methotrexate ADRs	Sarcoidosis-associated morbidity
Cardiovascular	Hypertension Coronary heart disease Ischaemic heart disease Heart failure	Pericardial serositis	Palpitations Heart rhythm disturbances Chest pains Heart failure Syncope
Dermatologic	Dermatoprosis Skin atrophy Ecchymosis Purpura Erosions Striae Delayed wound healing Easy bruising Acne Hirsutism Hair loss	Oral ulcers Alopecia Rash Anaphylactic reactions Photosensitivity Vasculitis Nodulosis	Macular or plaque skin lesions, especially over the face and hands, and involving tattoos Vitiligo
Endocrine and metabolic	Hyperglycemia Diabetes mellitus Dyslipidemia Weight gain Cushingoid features Growth suppression Adrenal suppression Secondary hyperparathyroidism		Hypothalamic-pituitary infiltration by sarcoid granulomata Hypogonadism Hypercalcemia
Gastrointestinal	Gastritis Peptic ulcer Gastrointestinal bleeding Visceral perforation Pancreatitis	Vomiting Diarrhoea Gastrointestinal bleeding Complication of ulcers	Gastrointestinal involvement
Hepatic	Hepatic steatosis	Elevation of liver enzymes Fibrosis Cirrhosis	Liver involvement Elevation of liver enzymes Fibrosis Jaundice Ascites Pruritis
Immunologic	Suppression of cell-mediated immunity Predisposition to infections Reactivation of latent infections	Suppression of cell-mediated immunity Opportunistic infections	Splenomegaly
Musculoskeletal	Osteoporosis Avascular necrosis of bone Myopathy	Osteopathy	Muscle weakness/atrophy Myopathy Adjacent joints and bones can be involved
Neurologic/ neuropsychiatric	Fatigue Cognitive impairment Mood alteration Depression Euphoria Irritability Akathisia Anxiety Psychosis Dementia Delirium	Fatigue Cognitive impairment Mood alteration Dizziness Headache Vertigo	Fatigue Cognitive impairment Mood alteration Depressive symptoms Headaches Seizures Cranial nerve deficits Anxiety Focal peripheral neuropathies Small fibre neuropathy
Ophthalmologic	Posterior subcapsular cataract Increased intraocular pressure Glaucoma Ptosis Mydriasis Opportunistic ocular infections Central serous chorioretinopathy	Conjunctivitis Blurred vision Photophobia Blepharitis Decreased reflex tear secretion Peri-orbital edoema Non-arteritic ischaemic optic neuropathy	Painful conjunctivitis Vision loss Photophobia Hyperemia Tearing eyes Floaters Blurring, gritty eyes
Renal		Renal insufficiency (only in pre-existing, severely impaired renal function)	Granulomatous interstitial nephritis Nephrocalcinosis Hematuria Nephrolithiasis
Respiratory	Obstructive sleep apnoea	Interstitial pneumonitis Pneumocystis Jirovecii pneumonia (PJP)	Obstructive sleep apnoea Various pulmonary manifestations Endstage pulmonary fibrosis
Urogenital		Abortion Malformation Defective oogenesis and spermatogenesis Gynaecomastia	Azoospermia Bladder dysfunction Multiple localizations female genitalia Granuloma in the breast

Second-line agents, such as MTX, an agent originally intended for antineoplastic use, be used as an alternative to GCs [38,164,165]. At a low dose, MTX acts as an anti-inflammatory agent and immunomodulator. The drug is a folate analog that antagonises the enzyme dihydrofolate reductase,[166] but MTX suppresses inflammation mostly by inhibiting adenosine deamination, and potentiates the adenosine‐induced vasodilatation [167]. Therefore, the supplementation of MTX therapy with folate is strongly recommended. Given its mechanism of action, MTX can cause many adverse events, including nausea, anorexia and headache (see Table 2) [157,168,169]. Moreover, MTX administration can increase the risk for drug-drug interactions and can expose the patients to a significant likelihood of MTX toxicity [165]. Laboratory abnormalities are rather non-specific and can include moderate leukocytosis, mild eosinophilia and elevation of lactate dehydrogenase (LDH). Although one of the main concerns of MTX use is hepatotoxicity, such as elevated transaminase levels and fibrosis, Baughman et al. found in a retrospective study including 607 sarcoidosis patients that MTX use was associated with a low incidence of hepatic or haematologic complications [164]. They concluded that MTX can be considered as a safe and effective treatment for sarcoidosis patients [164]. Moreover, the authors indicated that a close follow-up of treated patients with MTX [165], could be useful to detect liver and haematologic alterations at blood exams, and liver elastography could be indicated as method for monitoring liver toxicity such as steatosis or overt cirrhosis. Safety considerations include that the drug should be avoided in pregnant women as well as caution when it is administered with other drugs. Indeed, the use of high dose MTX along with nonsteroidal anti-inflammatory agents (NSAIDs) and/or antibacterial agents, such as penicillin, may cause myelosuppression, transaminitis and renal failure. However, the clinical importance of the interaction between low-dose MTX (7.5- 25 mg/day) and NSAIDs, penicillins, or proton pump inhibitors (PPIs) cannot be substantiated [170].

To date, in the treatment of sarcoidosis GCs and MTX are the two most commonly used drugs. Currently, the ongoing PREDMETH study - a randomised controlled trial - aims to investigate the effectiveness and tolerability of MTX compared with prednisone as first-line therapy in patients with pulmonary sarcoidosis [171]. The demonstration of a similar effectiveness, but with fewer ADRs, between MTX and prednisone as first-line treatment may lead to a significant change in clinical practice and improved patient care. This study will also allow gathering information about the prevalence of ADRs. The comorbidities of GCs and MTX, as well as sarcoidosis-associated morbidity are summarised in Table 2 [2,154,172].

Azathioprine (AZA) is a purine antagonist that is often used in the management of sarcoidosis when MTX is contra-indicated, such as in pregnancy, or has failed to induce significant improvement. AZA exerts an anti-inflammatory effect by reducing the proliferation of B- and T-cells. Most common ADRs include infections, gastro-intestinal complaints, hepatic function decline and bone marrow depression, but fever and fatigue may also occur. These latter symptoms can be hard to distinguish from sarcoidosis-associated symptoms, but their rapid resolution following drug discontinuation support an AZA-associated phenomenon. AZA is metabolised by thiopurine S-methyltransferase (TPMT), and patients with low TPMT levels can develop severe neutropenia. TPMT genotyping is therefore advised before starting treatment with AZA to reveal patients susceptible to toxicity [173].

Monoclonal antibodies targeting TNF-α, particularly infliximab and adalimumab, are recommended as third-line treatment. Most commonly reported ADRs include infections. Concomitant use of immunosuppressants such as MTX reduces the risk of neutralising antibodies against TNF-α inhibitors and increases drug efficacy [174]. A study in rheumatoid arthritis showed that adalimumab levels are influenced by concomitant MTX use: patients on adalimumab monotherapy had a lower adalimumab level, whereas patients concomitantly taking MTX had a higher level [174].

### ADRs of polypharmacotherapy of sarcoidosis

Beside the treatment of sarcoidosis polypharmacy by itself may have a substantial influence. Thus, drug-induced ADRs may cause additional comorbidities in patients with sarcoidosis [175]. Drug-induced interstitial lung disease (DI-ILD) can be suspected in a patient with sarcoidosis who develops cough, dyspnoea and clinical and pathological features that are compatible with ILD during treatment with certain drugs (soon after their introduction), or shows substantial deterioration of pre-existing symptoms. As these drug-induced manifestations can be hard to distinguish from those associated with sarcoidosis, their prevalence is likely to be underestimated. Consequently, only very few such cases have been reported [175,176].

Besides rare gastrointestinal (GI) manifestations of sarcoidosis (e.g. from the direct involvement of GI system from sarcoidosis lesions), some studies have indeed demonstrated that sarcoidosis is associated with an increased risk events such as upper GI ulcer and/or haemorrhage and diverticulitis. These manifestations, however, are more likely to be side effects of drugs such as NSAIDs and biologic agents. In particular, long-term use of NSAIDs to control chronic symptoms such as musculoskeletal pain, is associated with cyclooxygenase-1 (COX-1) inhibition, reduction of prostaglandins production and increased hazard of gastroduodenal ulcers. The observation that patients using biologic agents have the highest risk of upper GI events supports the hypothesis that dysregulation of the immune system represents a concomitant disease mechanism [177].

In summary, it is important to consider the option of drug-induced damage, especially as the clinical situation deteriorated after the introduction of a certain drug. Patients should be closely monitored for the development of potential ADRs or drug-induced comorbidities. The variability in drug response among patients including sarcoidosis is multifactorial. Therefore, increased recognition and a high index of suspicion are key to proper diagnosis and prompt withdrawal of the offending drug where the mainstay of treatment is drug cessation. Though, early recognising and managing drug-induced morbidity is a real clinical challenge. A tailored approach that can be adopted in clinical practice, based on the disease severity and risk profiles, is warranted. There is a need to develop trials investigating the role of genomic variations in not only disease susceptibility and predicting prognosis, but also treatment response, and in tailoring drug treatment for individual patients. Comorbidities or ADRs of drugs used in the treatment of sarcoidosis are sometimes very hard to differentiate from symptoms of sarcoidosis itself, which may lead to diagnostic issues.

## Conclusion

The study of the possible interactions between sarcoidosis and other comorbidities is a field of great interest. The coexistence of multiple conditions complicates the diagnosis of the disease and might be responsible, at least in part, for the highly variable natural history of sarcoidosis, particularly if more than one diseases is present. Therefore, a thorough evaluation of all disorders that may coexist with sarcoidosis should be carried out.

In particular, assessment organs at high risk of comorbidities, such as heart and lungs, is highly recommended. In turn, functional assessment of these major organs, if involved, should be performed in order to timely initiate appropriate treatment to mitigate disease manifestations and prevent long-term complications. Attention should also be paid to non-specific organ-related symptoms, such as fatigue and pain, which may be caused by adverse drug reactions rather than sarcoidosis. Future studies on sarcoidosis should specifically focus on the presence of comorbidities. In particular, population-based cohort studies should investigate the risk of comorbidities in patients with sarcoidosis, with the aim to predict the long-term risk of comorbidities and related outcomes.

## Data Availability

All figures and tables are original and are not taken from other publications. Data sharing is not applicable to this article, as no new data were created or analysed in this study.
